# Prof. Artur Beltrame Ribeiro: a life devoted to making new friends until the very end

**DOI:** 10.1590/2175-8239-JBN-2026-IM01en

**Published:** 2026-01-26

**Authors:** José Medina Pestana, Lúcio Requião-Moura

**Affiliations:** 1 Universidade Federal de São Paulo, Escola Paulista de Medicina, Divisão de Nefrologia, São Paulo, SP, Brazil.; 2 Hospital do Rim, Fundação Oswaldo Ramos, São Paulo, SP, Brazil.

With a heavy heart, we undertake the honorable task of paying tribute to the memory of our friend, colleague, mentor, and leader, Professor Artur Beltrame Ribeiro, who passed away on 30 September 2025 at the age of 80. Speaking about Artur is easy; what is difficult is condensing into a few lines the stature of the physician, professor, researcher, institution builder, and the loyal, discreet, and affectionate man who, throughout his life, had the gift of continually winning new friends. His life’s journey left a lasting mark on nephrology and hypertension in Brazil.

Artur graduated from the *Escola Paulista de Medicina* (EPM) in 1969. On his first day of medical school in 1964, he had an experience that would come to symbolize the years ahead: his inaugural class was, in fact, a student assembly convened to discuss the turbulent political moment the country was facing. From that point on, he became involved in student politics, eventually serving as president of the EPM Student Union. Even during the most repressive years of the military dictatorship, both in medical school and throughout his residency, he did not renounce his role as an active agent in the defense of social justice and democratic principles, maintaining close ties with leaders who would later play key roles in Brazil’s re-democratization.

In his professional and academic training, he completed a PhD in nephrology at EPM in 1977. He subsequently pursued postdoctoral studies at Cornell University, returning to Brazil to dedicate himself to academic medicine and excellence in public health. At EPM, where he became a full professor of nephrology, he stood out for his integrative vision, combining clinical nephrology, arterial hypertension, and translational research. His work transcended national boundaries, leading him to serve as a visiting professor at Boston University School of Medicine. In 2005, he received the IASH Lifetime Achievement Award, recognizing him as one of the leading figures in his field in the Americas.

A close disciple of Professor Oswaldo Ramos^1^, Artur played a central role in consolidating the *Fundação Oswaldo Ramos* and the *Hospital do Rim*, institutions he helped transform into national and international references in kidney transplantation, high-complexity care, workforce training, and scientific production. As a leader of the *Fundação Oswaldo Ramos* from its inception, he shared with Professor Medina the mission of imprinting enduring hallmarks on the institution: rigorous oversight of public resources; exemplary integration of care, teaching, research, and extension; and the consolidation of the *Hospital do Rim* as one of the main cornerstones in the training of nephrologists for Brazil, Latin America, and many other countries across all continents^2,3^. Artur and Medina became close friends, and since then, their families have enjoyed a warm and harmonious relationship.

Within professional societies, Artur projected Brazilian nephrology and hypertension far beyond our borders. He played a prominent role in the Brazilian Society of Nephrology (BSN), was a founder and the first president of the São Paulo State Society of Nephrology (SONESP), and served as president of the Brazilian Society of Hypertension (SBH). He collaborated with international organizations, including the International Society of Hypertension (ISH). Furthermore, he served as president of the InterAmerican Society of Hypertension (IASH), consistently defending evidence-based medicine, scientific rigor, and social responsibility.

Consistent with this vision, his name is associated with research projects that influenced the development of therapeutic regimens for the treatment of arterial hypertension, extending his impact internationally. These efforts translated into high-impact scientific publications, participation in guideline development, continuing medical education programs, and initiatives that helped train generations of clinicians and nephrologists^4,5^. His scientific and institutional contributions were recognized with distinctions of great national significance, such as the National Order of Scientific Merit, as well as his election to the Brazilian Academy of Sciences.

For those who lived and worked with him, however, his most valued distinction was expressed day after day: the trust of patients, colleagues, trainees, nurses, technicians, and staff, who found in Artur firm principles, attentive listening, sound guidance, and constant loyalty. He was demanding and precise in clinical reasoning, yet never lost his gentleness in human interactions. He championed technical excellence, continual updating, and disciplined study, but taught with calmness and serenity. Many who today lead nephrology and hypertension services across Brazil recognize him as the mentor who opened doors, cultivated vocations, and generously shared opportunities. His legacy is embodied in the dozens of disciples who became leaders in medical schools throughout the country.

As we honor the memory of Professor Artur, we do more than record dates, positions, and titles. We celebrate the educator who trained generations; the clinician who honored the trust of his patients, from the most modest citizen to ministers of state or president of the republic; the leader who helped build and sustain the fundamental pillars of Brazilian nephrology; and the continuer and, at the same time, the creator, who knew how to transform admiration into concrete and lasting work. We remember the fisherman who saw, in fishing trips, one of his favorite social activities, an opportunity to strengthen bonds of friendship. Not only that, but we also honor the serene and loyal friend who built and maintained friendships throughout his life.

To his family, whom he loved deeply and with whom he discreetly shared the weight of his public responsibilities, we express, on behalf of our community, our solidarity and gratitude. To the community of nephrologists, cardiologists, and hypertension specialists in Brazil and abroad, the commitment is to preserve and ensure that his example continues to flourish. On behalf of the Brazilian Journal of Nephrology and the community he helped to build, we record our respect and our tribute to Artur Beltrame Ribeiro, mentor, leader, friend, and moral and intellectual reference, whose absence will be deeply felt, but whose presence will remain alive in our daily practice, in the institutions he helped to strengthen, and in the lives he transformed.
