# Supplementary material for: Prof. Artur Beltrame Ribeiro: a life devoted to making new friends until the very end
Source: J Bras Nefrol. 2026 Jan 26;48(1):e2026IM01. doi: 10.1590/2175-8239-JBN-2026-IM01en (PMC12959924; doi:10.1590/2175-8239-JBN-2026-IM01en)
Supplement: Translation

# Translation: Prof. Artur Beltrame Ribeiro: uma vida dedicada a conquistar novos amigos até o fim

**DOI:** 10.1590/2175-8239-JBN-2026-IM01pt

**Authors:** José Medina Pestana, Lúcio Requião-Moura

**Affiliations:** 1 Universidade Federal de São Paulo, Escola Paulista de Medicina, Divisão de Nefrologia, São Paulo, SP, Brasil.; 2 Hospital do Rim, Fundação Oswaldo Ramos, São Paulo, SP, Brasil.

Cumprimos, com profundo pesar, a honrosa missão de saudar a memória de nosso amigo, colega, mestre e líder, Professor Artur Beltrame Ribeiro, que nos deixou em 30 de setembro de 2025, aos 80 anos. Falar de Artur é fácil; difícil é condensar, em poucas linhas, a grandeza do médico, do professor, do pesquisador, do construtor de instituições e do homem leal, discreto e afetuoso que, ao longo de toda a sua vida, manteve a virtude de sempre conquistar novos amigos. Sua trajetória de vida marca, de modo definitivo, a Nefrologia e a Hipertensão no Brasil.

Formado em 1969 pela Escola Paulista de Medicina (EPM), Artur teve, já em seu primeiro dia de aula, em 1964, uma experiência que simbolizaria os anos seguintes: sua aula inaugural foi, na realidade, uma assembleia estudantil destinada a discutir o conturbado momento político vivido pelo país àquela época. A partir dali, envolveu-se na política estudantil, chegando à presidência do Centro Acadêmico da EPM. Mesmo diante das investidas contra sua liberdade nos períodos mais duros da ditadura militar, tanto na graduação quanto na residência, não renunciou ao papel de agente ativo na defesa da justiça social e dos princípios democráticos, mantendo proximidade com lideranças que seriam fundamentais no processo de redemocratização do país nas décadas seguintes.

Em sua formação profissional e acadêmica, concluiu o doutorado em Nefrologia pela EPM em 1977 e realizou pós-doutorado na *Cornell University*, retornando ao Brasil para se dedicar à medicina acadêmica e à saúde pública de excelência. Na EPM, onde se tornou Professor Titular da Disciplina de Nefrologia, destacou-se por uma visão integradora entre nefrologia clínica, hipertensão arterial e pesquisa translacional. Sua atuação ultrapassou fronteiras, tendo se tornado Professor Visitante da *Boston University School of Medicine*. Em 2005, recebeu o *IASH Lifetime Achievement Award*, em reconhecimento à sua estatura como um dos principais líderes das Américas em sua área.

Discípulo próximo do Professor Oswaldo Ramos^1^, Artur assumiu papel central na consolidação da Fundação Oswaldo Ramos e do Hospital do Rim, instituições que ajudou a transformar em referências nacionais e internacionais em transplante renal, assistência de alta complexidade, formação de recursos humanos e produção científica. Como líder da Fundação Oswaldo Ramos desde o início de suas atividades, dividiu com o Prof. Medina a missão de implementar marcas sólidas na instituição: rigor na gestão dos recursos públicos; integração exemplar entre assistência, ensino, pesquisa e extensão; e a consolidação do Hospital do Rim como uma das principais forças motrizes na formação de nefrologistas para o Brasil, para a América Latina e diversos países em todos os continentes^2,3^. Artur e Medina tornaram-se grandes amigos na vida pessoal e, desde então, suas respectivas famílias desfrutam de convívio fraternal e harmonioso.

Na vida associativa, Artur projetou a Nefrologia e a Hipertensão brasileiras para além de nossas fronteiras. Teve atuação destacada na Sociedade Brasileira de Nefrologia (SBN), foi fundador e primeiro presidente da Sociedade de Nefrologia do Estado de São Paulo (SONESP) e presidiu a Sociedade Brasileira de Hipertensão (SBH). Colaborou com entidades internacionais, como a *International Society of Hypertension* (ISH), além de exercer a presidência da *InterAmerican Society of Hypertension* (IASH), sempre em defesa da medicina baseada em evidências, do rigor científico e da responsabilidade social.

Coerente com essa visão, seu nome está ligado a projetos de pesquisa que influenciaram o desenvolvimento de esquemas terapêuticos para o tratamento da hipertensão arterial, ampliando sua relevância internacional. Esses esforços se traduziram em publicações científicas de impacto, participação na elaboração de diretrizes, programas de educação continuada e iniciativas que ajudaram a formar gerações de clínicos e nefrologistas^4,5^. Sua contribuição científica e institucional foi reconhecida com distinções de grande significado nacional, como a Ordem Nacional do Mérito Científico e o assento na Academia Brasileira de Ciências.

Para os que com ele conviveram, porém, sua maior condecoração era cotidiana: a confiança irrestrita de pacientes, colegas, alunos, enfermeiros, técnicos e colaboradores, que encontravam em Artur firmeza de princípios, escuta atenta, orientação segura e lealdade inabalável. Era exigente e preciso no raciocínio clínico, sem nunca perder a delicadeza no trato humano. Defendia a excelência técnica, a atualização permanente e o estudo disciplinado, mas ensinava com serenidade. Muitos dos que hoje lideram serviços de Nefrologia e Hipertensão no Brasil reconhecem nele o mestre que abriu portas, estimulou vocações e compartilhou generosamente oportunidades. Seu legado se faz presente na formação de dezenas de discípulos que se tornaram líderes em diversas escolas médicas em todo o país.

Ao despedirmo-nos do Professor Artur, não nos limitamos a registrar datas, cargos ou títulos. Celebramos o educador que formou gerações; o clínico que honrou a confiança de seus pacientes, do mais simples cidadão até Ministros de Estado e Presidente da República; o líder que ajudou a erguer e sustentar pilares fundamentais da Nefrologia brasileira; e o continuador e, ao mesmo tempo, o criador, que soube transformar admiração em trabalho concreto e duradouro. Celebramos também o pescador que via nos grupos de pescaria, uma das suas atividades sociais preferidas, uma oportunidade para fortalecer laços de amizade; e o amigo sereno e leal que construiu amizades até o fim de sua vida.

À sua família, a quem tanto amou e com quem compartilhou, com discrição, o peso de suas responsabilidades públicas, expressamos, em nome da nossa comunidade, solidariedade e gratidão. À comunidade de nefrologistas, cardiologistas e especialistas em hipertensão arterial do país e do exterior, fica o compromisso de preservar e fazer frutificar seu exemplo. Em nome do *Brasilian Journal of Nephrology* e da comunidade que ele ajudou a construir, registramos nosso respeito e nossa homenagem a Artur Beltrame Ribeiro, mestre, líder, amigo e referência moral e intelectual, cuja ausência se fará sentir profundamente, mas cuja presença permanecerá viva em nossa prática diária, nas instituições que ajudou a fortalecer e nas vidas que transformou.
